# Effects of a Combination of Lysolecithin, Synthetic Emulsifier, and Monoglycerides on Growth Performance, Intestinal Morphology, and Selected Carcass Traits in Broilers Fed Low-Energy Diets

**DOI:** 10.3390/ani11113037

**Published:** 2021-10-22

**Authors:** Abdallah Ghazalah, Mamdouh Abd-Elsamee, Moataz Ibrahim, Sherein S. Abdelgayed, Mohamed Abdelkader, David Gonzalez-Sanchez, Alexandra Wealleans

**Affiliations:** 1 Department of Animal Production, Faculty of Agriculture, Cairo University, Giza 12613, Egypt; ghazalah@gmail.com (A.G.); mamdouh20466@yahoo.com (M.A.-E.); mmibrahim@cpg.com.eg (M.I.); 2 R&D Department, Feed Division, Cairo Poultry Company, Giza 12613, Egypt; 3 Department of Pathology, Faculty of Veterinary Medicine, Cairo University, Giza 12613, Egypt; Sherein.abdelgayed@vet.cu.edu.eg; 4 Kemin Animal Nutrition and Health, 2200 Herentals, Belgium; mohamed.abdel-kader@kemin.com (M.A.); Alexandra.Wealleans@kemin.com (A.W.)

**Keywords:** broiler, carcass trait, gut morphology, lysolecithin, performance

## Abstract

**Simple Summary:**

Lysolecithin is produced from the enzymatic conversion of lecithin, resulting in a smaller molecule better able to improve the process of digestion of fats and oils than its progenitor. In broiler production, lysolecithin can improve performance when added to nutritionally adequate diets, but also when diets are reformulated to provide lower levels of energy and amino acids. Low-energy diets may provide more ‘space’ for growth improvements, but there is a scarcity of data on the effect of lysolecithin when added to low-energy diets containing only intact fat from raw feed ingredients. Moreover, the ability of pure lysolecithin to improve energy digestion and absorption can be further improved by the addition of synthetic emulsifiers and monoglycerides. The present study aims to evaluate the effect of supplementing a combination of lysolecithin, synthetic emulsifier, and monoglycerides on growth performance, intestinal morphology, carcass traits, and meat characteristics in broilers fed commercially relevant low-energy diets without added oil. The results revealed that this combination could effectively improve growth performance, carcass characteristics, and intestinal morphology of broiler chickens.

**Abstract:**

This study aimed to evaluate the effect of supplementing a combination of lysolecithin, synthetic emulsifier, and monoglycerides (LEX) on growth performance, intestinal morphology, and selected carcass traits in broilers fed low-energy diets without added oil. Three hundred one-day-old Arbor Acres (AA) broilers (40.3 ± 3.3 g) were assigned to two dietary treatments with six replicates of 25 birds each and were fed a control low-energy diet without added oil supplemented with 0 and 250 g/t of LEX for 30 days. Growth performance was measured and recorded throughout the study. At slaughter, 60 birds per treatment were used to assess the effect of LEX on the carcass traits. Final average body weight and feed conversion ratio were improved (*p* < 0.05) in LEX treated birds compared to control. LEX supplementation was linked to higher (*p* < 0.05) carcass weight and yield and to lower (*p* < 0.05) abdominal fat and liver weight. Moisture content was higher (*p* < 0.05) in ground deboned broilers from LEX treatment. Villus height was increased (*p <* 0.05), and crypt depth reduced (*p <* 0.05) in the jejunum of birds treated with LEX. This study demonstrates that supplementation of LEX to a low-energy diet without added oil improved performance, carcass weight and yield, reduced abdominal fat deposition, and improved intestinal morphology in broiler chickens.

## 1. Introduction

Modern fast-growing broilers have high energy and amino acid requirements [1,2] and meeting these requirements leads to improved feed efficiency, but also increases diet costs, especially when the price of oils and protein meals is volatile. Increasing the utilization of dietary energy and other nutrients can allow nutritionists to formulate diets to lower basal nutrient levels and lower feed costs. Among other approaches to reformulate diets to lower nutrient density, such as non-starch polysaccharide degrading enzymes, proteases or phytases, the use of lysolecithin-based products has received both academic [3] and commercial attention [4].

Lysolecithin is produced from the enzymatic conversion of lecithin, cleaving one of the fatty acids from the glycerol backbone of the phospholipids [5]. The resulting lysophospholipids are smaller and more hydrophilic molecules, with a lower critical micelle concentration than phospholipids [6]. This improves their emulsifying properties in oil-in-water environments, thus improving the process of digestion of fats and oils [7]. In broiler production, the addition of lysolecithin has been shown to improve the availability of energy [8,9,10]—driven by improvements in the hydrolysis and absorption of fatty acids [7,11] from both added fats and cereal grains—and other nutrients including protein and amino acids [11,12]. The ability of pure lysolecithin to improve energy digestion and absorption can be further improved by the addition of synthetic emulsifiers and monoglycerides [3]. At the same time, dietary lysolecithin interacts with birds’ physiology, including the incorporation of lysophospholipids into the epithelial cell walls [13,14] and epigenetic interactions [15], leading to improved gut function and structure [16]. This leads to improved weight gain and feed efficiency [4,7,8,10,17,18], as well as reductions in fat deposition and related improvements in carcass yield [16].

Wealleans et al. [4] showed that the supplementation of lysolecithin to broiler diets can improve performance when added on top of nutritionally adequate diets, but also when diets are reformulated to provide lower levels of energy and amino acids. Similar improvements in the performance of diets reduced in energy were also reported by Zhao and Kim [19], Boontiam et al. [20], Chen et al. [16], and Haetinger et al. [10].

Dietary energy is, together with amino acids, the most expensive nutrient in poultry diets [21]. Rising or volatile costs of feed ingredients, especially fats and oils, mean that minimum dietary energy requirements can be challenged in feed formulation, which may result in the full disappearance of added oil from the diets and a subsequent loss of growth performance. These types of low-energy diets may provide more space for growth improvements when supplemented with lysolecithin. However, there is a scarcity of data on the effect of lysolecithin when added to low-energy diets containing only intact fat from raw feed ingredients (with no added fat). Therefore, this study aimed to evaluate the effect of supplementing a combination of lysolecithin, synthetic emulsifier, and monoglycerides on growth performance, intestinal morphology, and selected carcass traits in broilers fed low-energy diets without added oil.

## 2. Materials and Methods

### 2.1. Broilers, Diets, and Housing

The trial was conducted at the Broiler Research Unit of Cairo Poultry Company. All experimental procedures were in line with commercial practices and approved by the institutional animal care and use committees of the Faculty of Science, Cairo University (CUIIF2420) and were compliant with all local animal welfare legislation. The duration of the study was 30 days. A total of three hundred one-day-old Arbor Acres (AA) broilers (40.3 ± 3.3 g at hatch) were sourced from the commercial hatchery of Cairo Poultry Company. Birds were feather sexed on the day of hatch and, upon arrival at the trial site, were randomly allocated to two dietary treatments with six replicates of 25 mixed-sex broilers (13 males and 12 females) each: (i) a control low-energy diet (ii) the same low-energy diet supplemented with a combination of lysolecithin, synthetic emulsifier and monoglycerides (LEX) at 250 g/t in the expense of 250 g/t of corn. The combination of lysolecithin, synthetic emulsifier and monoglycerides used in this study was LYSOFORTE^®^ Extend and was supplied by Kemin Europa NV, Herentals, Belgium.

Birds received all standard hatchery vaccinations (Newcastle Disease, Infectious Bronchitis, IBD, H5N1), and no concomitant drug therapy was used during the study. Pens were of equal size (2 m^2^), and pen allocation per treatment was randomized. Pens had a solid floor and used clean wood shavings as litter material. The temperature and ventilation of the building were monitored daily and were optimal for the age of the birds according to the breed recommendations. A regular lighting program (0–3 days 24 h/light, 4–7 days 23 h/light, and 8–final age 20 h/light) was provided by fluorescent bulbs placed above the pens.

Diets were fed in three phases, with a starter diet from 0–10 days, a grower diet from d11–21 days, and a finisher diet from 22–30 days. Diets were formulated to low-energy content compared to Arbor Acres broiler nutrition specifications [22] with 100, 150, and 200 kcal/kg lower metabolizable energy (ME) for starter, grower, and finisher feeding phases respectively. Nutrient values of the diets were calculated according to the values of feedstuffs used by Cairo Poultry Company. All diets were produced according to commercial practices and fed as pellets (2 mm diameter and 3 mm length in starter diet; and 3 mm diameter and 5 mm length in grower and finisher diets). The composition of the experimental diets is shown in Table 1. Feed and water were provided ad libitum throughout the study.

### 2.2. Growth Performance Evaluation

All birds were weighed individually after their arrival from the hatchery and at days 7, 14, 21, 28, and 30. Individual weights were averaged to provide pen-level data. Feed bags, as well as feed remaining in the feeders, were weighed at the same time and these values were used to calculate feed intake and feed conversion ratio (FCR). Pens were monitored daily for mortality. On day 30, all birds were slaughtered and final bodyweight gain, feed intake, and FCR were calculated.

### 2.3. Carcass Traits and Meat Characteristics

At slaughtering, 60 birds per treatment (10 birds from each replicate: 5 males and 5 females) with an average body weight of 1700 g were selected and euthanized for the carcass traits evaluation. This selection protocol was conducted to avoid differences in carcass yield created by differences in final live bodyweight. Birds were slaughtered following the Halal method (Islamic method as per Egyptian law), through a cut to the jugular vein, carotid artery and windpipe. After slaughtering and bleeding the carcasses were de-feathered and eviscerated by hand and individual carcass was weighed. The carcass was cut into parts and the following traits were weighed: wings, fillets, tender, whole breast, drums, thighs, skin, abdominal fat, gizzard, heart, and liver. All the carcass traits were expressed as a percentage of the slaughter weight; except the abdominal fat, which was expressed as a percentage of the carcass weight.

Breast, thigh, and drumstick samples were taken from the eight chicken carcasses per treatment (four males and four females) and were deboned, skinned and superficial fat was removed. Subsequently, each set was ground and homogenized for determination of moisture and fat content (AOAC, 2006; Method 950.46 and 960.39 respectively) [23], which were expressed as a percentage of the analyzed sample.

### 2.4. Intestinal Morphology

At slaughtering, one bird from each replicate (three males and three females in total from each treatment) was euthanized and specimens from the jejunum were collected and fixed in 10% formal saline, then washed, dehydrated, cleared, and embedded in paraffin. As described by Bancroft and Stevens [24], the paraffin-embedded blocks were sectioned at 4–5 μm thickness, stained with hematoxylin and eosin and examined for the morphometric changes in the villi height and crypts depth using a digital microscope (Olympus BX50, Japan) and calibrated TSView software. Two measurements of villus height (VH) and crypt depth (CD) were made from each slide, and the average value of these measurements was used for statistical analysis. The ratio of villus height: crypt depth (VH:CD) for each replicate was calculated from the average measurement.

### 2.5. Statistical Analysis

The pen/replicate was considered the experimental unit for performance. The individual broiler sampled was considered the experimental unit for carcass traits and meat characteristics and histology. No outlier data was identified or excluded from the dataset. Performance, selected carcass traits, meat characteristics, and histology data were analyzed using one-way ANOVA with JMP 15 (SAS Institute, Cary, NC, USA), with LEX supplementation as the main factor. Data are presented as means with overall standard error of the mean (SEM). In all analyses, differences were considered significant at *p* < 0.05.

## 3. Results

### 3.1. Growth Performance

Table 2 presents the effect of dietary supplementation of LEX to low-energy diets on growth performance in broilers of each experimental group measured at different growth stages. There was no mortality throughout the trial for both treatments. There was no difference (*p* = 0.6753) in bird weight between groups at the start of the study, with chicks weighing an average of 40.3 ± 3.3 g at day 0. The body weight (BW) at hatch was 2.7 g below the standard of 43 g provided by the genetic company [25]. On day 14, the BW of birds from both treatments was well above the performance objective of 512 g [25], showing no influence of the lower BW at hatch on future growth performance compared to the genetic standards. By day 14, there was a tendency towards increased (*p* = 0.0752) BW for the birds receiving the LEX compared to the control. There was no difference (*p* = 0.6921) in feed intake between treatments at day 14, however, between days 7 and 14 there was an improvement (*p* = 0.0228) in FCR for the LEX treatment compared to the control. Between days 14 and 21 there was an increase (*p* = 0.0016) in the BWG and feed intake (*p* = 0.0246) for the LEX treatment compared to the control. On day 21, BW was higher (*p* = 0.0007) for the LEX birds compared to the control. BW at 28 days as well as at catch (day 30) continued to be higher (*p* < 0.05) for birds fed the LEX-supplemented diets compared to the control. Over the course of the entire trial, birds fed the LEX-supplemented diets showed better (*p* = 0.0027) FCR than those fed the control diet.

### 3.2. Carcass Traits and Meat Characteristics

Table 3 presents the effect of dietary supplementation of LEX to low-energy diets on slaughter weight and carcass traits of broilers in each experimental group. As broilers were selected to have a similar average slaughter weight (around 1700 g) between both treatments, this parameter was therefore not different (*p* = 0.4240). However, carcass weight as well as carcass yield (expressed as a percentage of the slaughter weight), were higher (*p* = 0.006 and *p* = 0.0431, for carcass weight and carcass yield respectively) for the LEX treatment compared to the control. Wing percentage increased (*p* = 0.0123) for the LEX treatment compared to the control. The abdominal fat percentage (expressed as a percentage of the carcass weight) and liver weight were both reduced (*p* = 0.0121 and *p* = 0.0040, for abdominal fat and liver weight respectively) in the LEX-fed birds compared to the control. No effects (*p* > 0.05) were detected for the rest of the carcass traits evaluated.

Table 4 presents the effect of dietary supplementation of LEX to low-energy diets on the fat and moisture content of ground deboned breast, thigh, and drumstick of broilers in each experimental group. The LEX treatment demonstrated increased (*p* = 0.0078) moisture in the ground deboned meat and no difference (*p* = 0.1390) of fat content compared to the control.

### 3.3. Intestinal Morphology

**Figure 1:**
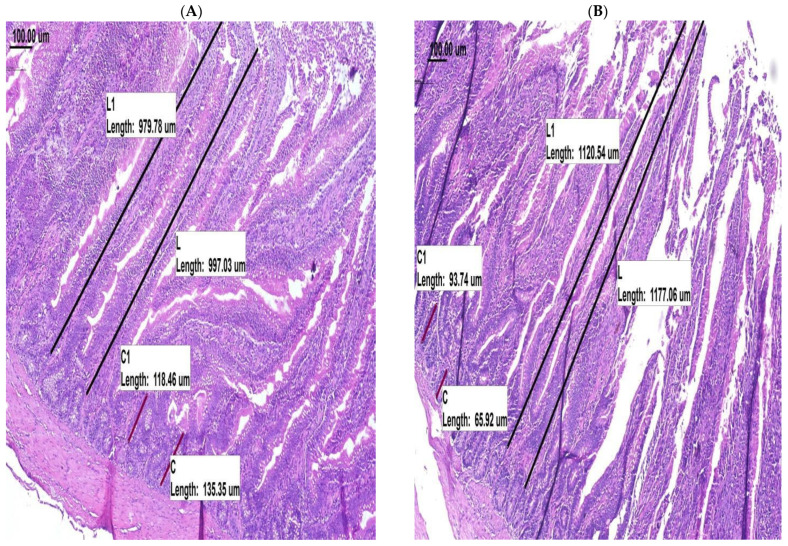
Morphological effects of LEX supplementation on broiler chicken jejunum. Treatments: (**A**) control, (**B**) LEX. ‘L’ and ‘C’ on the slides denote villus and crypt measurements, respectively; two measurements of each were taken per slide.

The morphometric changes in the jejunal villi of birds from control and supplemented groups are presented in Table 5 and Figure 1. In all replicate pairs, villus height was higher in treated birds than the control birds. Overall, supplementation with LEX led to longer (*p* = 0.0021) villus height and shorter (*p* = 0.0025) crypt depths compared to the control. The changes in villus height and crypt depth drove large differences in VH:CD ratios between treatments, with birds fed the control diet showing a lower (*p* = 0.0003) VH:CD ratio compared to the LEX supplemented birds.

## 4. Discussion

### 4.1. Growth Performance

Diet density has been found to be positively correlated with growth performance [26,27] and several previous research studies have shown impaired growth performance following dietary energy reductions [9,10,16,19,28,29]. Growth performance improvements following the supplementation of lysophospholipids-based products to broiler diets have been previously reported [4,9,10,11,19]. These improvements are associated with improved nutrient digestibility at early growing stages [11,19], as well as at later growing stages [9], and are also likely due to improved intestinal morphology [15,16]. Chen et al. [16] found that the lowest dose of lysolecithin 250 g/t from all tested doses (besides 500 and 750 g/t) was better for the growth performance when applied to reduced energy diets and concluded that supplementation dose should then be recommended according to dietary energy levels. However, none of these previous research studies evaluated lysolecithin supplementation to diets without added oil (only containing intact fat from feed ingredients). Siyal et al. [30], concluded that exogenous nutritional emulsifiers, such as lysolecithin, can help in fatty acid digestion, especially with poorly digestible fats and high inclusion rates of fats in the diet. Based on these conclusions, it could be hypothesized that lysolecithin application would not improve performance in low-energy diets with a low level of fat or without any added fat, especially if this fat profile is highly digestible. However, in several studies where lysolecithin was applied to low-energy diets, it has proven to improve growth performance [16,19,20]. Similarly, in our study, the supplementation of a combination of lysolecithin, synthetic emulsifier and monoglycerides to a low-energy broiler diet without added oil resulted in higher BWG and better FCR from hatch to catch compared to the same low-energy control diet. These improvements might be explained by a lower fat digestibility of intact sources of fat from feed ingredients compared to the same fat extracted from feed ingredients and subsequently added to the diet [31,32,33]. Part of this intact fat is bound or encapsulated in cell membranes or linked to fiber compounds in the raw materials, thus being less digestible [31]. It could be hypothesized that the diets without added oil used in this trial might have benefited more from LEX supplementation. However, fat digestibility was not evaluated in this study; therefore, the validation of this hypothesis would require further research.

### 4.2. Carcass Traits and Meat Characteristics

The value of carcass yield is of great economic importance and a key performance indicator in chicken slaughterhouses. It is becoming increasingly important, mainly because of the increase in portioned products produced in today’s meat processing industries. Previous studies where pure lysolecithin was used [8,16,34] did not report any significant difference for carcass yield. In two recent studies, from Thornhill [35] and Haetinger et al. [10] the supplementation of 500 g/t of the same combination of lysolecithin, synthetic emulsifier, and monoglycerides used in this study did not result in improvements of carcass weight nor yield. However, in this study, carcass yield was significantly improved in LEX treated birds. The main difference between these previous research experiences and the present trial, besides the composition of the diets, was the slaughtering weight and age of the broilers, both of them being substantially lower in this trial (2518 g, 35 days in Thornhill [35] and 3464 g, 42 days in Haetinger et al. [10] vs. 1700 g, 30 days in our study). It could be hypothesized that lower slaughtering weights might influence potential improvements of lysolecithin supplementation in broiler diets. However, the validation of this hypothesis would require further research and exploration of the specific mechanism behind it.

In modern genetic lines, selection for fast growth and efficient assimilation of amino acids into muscle has meant that there tends to be a positive correlation between dietary energy and amino acid content and breast (pectoralis major) yield [36], while fat pad deposition increases [1,2]. Although several authors investigated the effect of lysophospholipids (LPL) on carcass traits [8,16,29,34,37,38] improvements in breast meat yield have only been reported by Chen et al. [16]. This improvement was inconsistent, with an increase in % of pectoralis major only observed in birds fed high energy diets supplemented with 0.05% and 0.075% of LPL (but not with 0.025%) while not in birds fed the reduced energy diets at any LPL doses tested. Improvements in carcass yield in poultry can be associated with dietary increments in metabolizable energy, crude protein, and amino acids [39,40]. Several studies have shown lysolecithin supplementation to improve the apparent metabolizable energy (AME) of broiler diets [4,10,41] as well as nitrogen retention [9,10,19]. On the other hand, dietary energy level is a key factor to modify abdominal fat deposition. Increasing levels of diet density have been associated with higher abdominal fat deposition and there are several studies that found a reduction of abdominal fat deposition following dietary energy reductions [42,43,44,45]. Likewise, increased feed intake may result in caloric overconsumption, the excess of which could be deposited as fat. However, in this study, where low-energy diets were used, the supplementation of LEX significantly reduced abdominal fat pad and tended to increase breast meat yield. The effect of lysolecithin supplementation on the reduction of the abdominal fat pad is more consistently reported [16,19,46,47], even in low-energy diets and short fattening periods such as those of this study. Although nutrient digestibility was not assessed in this study, it could be hypothesized that supplementation of LEX may have improved nutrient digestibility and their utilization was diverted to growth and meat production rather than to inefficient metabolic processes involved in fat deposition. A similar mechanism was hypothesized by Boontian et al. [12], suggesting that lysophospholipids could change the utilization pattern of lipids and protein in the circulation, increasing their use for muscle formation instead of abdominal fat, therefore affecting protein and fat deposition in meat.

The selection for fast growth has also aggravated the burden on the liver, raising the risk of metabolic disorders, including fatty liver disease. Fat deposition in the liver can be encouraged by high levels of saturated dietary fat [48,49], but also by amino acid imbalances, especially that of methionine [1,2]. In our study, a significant reduction of liver weight was observed following LEX supplementation, which could be explained—though liver fat content was not measured directly—by a reduction in fat deposition and a reduced chance of fatty liver disease. Deposition of abdominal fat is a highly inefficient metabolic process and therefore the reduction in liver size and fat pad identified in the current study might be explained by more efficient digestion and absorption of fat in the LEX treated birds compared to those fed the control diets. The digestibility along with carcass traits evaluation should be evaluated in future trials to validate this hypothesis.

### 4.3. Intestinal Morphology

Increased jejunal villus height indicates a greater capacity for absorption of energy from fat, as the jejunum is the site of up to 82% of total fatty acid absorption [50]. Our results showed that LEX supplementation resulted in longer villus height and reduced crypt depth in the jejunum. These results support several other studies that report changes in intestinal morphology following lysolecithin supplementation. Khonyoung et al. [51] and Papadopoulos et al. [9] both reported that dietary lysolecithin increased mucosal height, while Boontiam et al. [12] and Chen et al. [16] showed that lysophospholipids supplementation of low-energy and low nitrogenous diets caused an increased jejunal villus height and a diminished crypt depth in the duodenum.

These changes may be driven by differences in the physical characteristics of the digestive content in the LEX treated group. Lysolecithin supplementation encourages the formation of smaller emulsion droplets and faster micelle creation [52]. As fat is emulsified faster, the matrix of fat surrounding other diet components is reduced, leading to lower reported ileal viscosity in lysolecithin supplemented birds [9]. However, the improvements in villus height in supplemented birds may also be partially due to more complicated physiological interactions between the lysolecithin and the bird itself. Lysolecithin can become incorporated in the epithelial cell walls [13,14], leading to greater flexibility and ease of nutrient transport, but may also exert epigenetic effects on the deposition of collagen in the villi, increasing their strength and height [15]. Although the effect of lysolecithin supplementation on added fat has mostly been investigated, it is clear from this study that these modes of action also applied to intact fat from feed ingredients. These improvements in gut structure and function allow better utilization of the available dietary nutrients, including energy [7,10,11,53] and protein [10,17,19], which subsequently may drive the improvements in performance efficiency and carcass yield reported in this study.

## 5. Conclusions

In summary, the use of a combination of lysolecithin, synthetic emulsifier, and monoglycerides proved to be effective in improving growth performance despite the absence of added fat in the diet. Furthermore, dietary supplementation of this combination showed a positive effect on intestinal morphology, improved carcass weight and yield, and reduced abdominal fat deposition. Therefore, the use of LEX could be beneficial to broiler producers using low-energy diets formulated without added oil whilst striving to achieve high growth performance standards and slaughtering yields.

## Figures and Tables

**Table 1:** Ingredients and nutrient composition of the experimental diets ^1^.

	0–10 Days	11–21 Days	22–30 Days
Ingredients (g/kg)			
Corn	545.7	567.0	619.6
Soybean meal, 47%	359.7	330.0	291.1
Extruded full-fat soybeans ^2^	53.3	77.0	70.0
Limestone	12.2	11.2	9.4
Corn gluten meal, 60%	10.0	-	-
Monocalcium phosphate	7.1	4.6	3.1
Sodium chloride	2.5	2.5	2.5
L-Lysine HCl	3.0	1.9	-
DL-Methionine	2.6	2.2	1.8
L-Threonine	1.7	1.4	0.3
Vitamin and mineral premix ^3^	2.0	2.0	2.0
NSP enzyme ^4^	0.1	0.1	0.1
Phytase ^5^	0.1	0.1	0.1
Calculated nutrient composition (%, as fed basis)			
Dry matter	88.06	87.97	87.84
ME, kcal/kg	2900	2950	3000
Crude protein	24.00	22.92	21.00
Crude fat	3.61	4.07	4.04
Crude fibre	2.77	2.86	2.56
Lysine	1.43	1.29	1.13
Methionine	0.58	0.52	0.50
Methionine + cysteine	0.88	0.82	0.84
Threonine	0.94	0.88	0.84
Arginine	1.42	1.37	1.38
Tryptophan	0.25	0.25	0.25
Ca	0.96	0.88	0.80
Available phosphorous	0.48	0.43	0.40
Na	0.16	0.16	0.16
Cl	0.23	0.21	0.18

^1^ To create the experimental treatment (LEX), LYSOFORTE^®^ EXTEND (a combination of lysolecithin, synthetic emulsifier, and monoglycerides manufactured by Kemin Europa NV, Herentals, Belgium) was added at 250 g/t at the expense of corn. ^2^ Trypsin inhibitors, 3.5 mg/g and Urease activity, 0.17 pH units. Production process consisting on pre-conditioning at 90 degrees Celsius for 120 s and extrusion at 135 degrees Celsius for 10 s. ^3^ Provided per kilogram diet: retinyl acetate, 3.44 mg; cholecalciferol, 0.088 mg; α-tocopherol acetate, 20 mg; menadione, 2.5 mg; thiamine, 2.0 mg; riboflavin, 6.5 mg; pyridoxine, 3.0 mg; cobalamin, 0.016 mg; niacin, 45 mg; calcium pantothenate: 13 mg; choline, 270 mg; Cu (from copper sulphate), 8.0 mg; Fe (from ferrous sulphate), 33 mg; I (from potassium iodate): 1.1 mg; Mn (from manganese sulphate), 90 mg; Se (from sodium selenite), 0.34 mg; Zn (from zinc oxide), 75 mg. ^4^ Protease 40,000 U/g, Xylanase 20,000 U/g and Amylase 2000 U/g. Axtra^®^ XAP 101 TPT. Danisco Animal Nutrition, DuPont. ^5^ 6-Phytase 10,000 FTU/g. Axtra^®^ PHY. Danisco Animal Nutrition, DuPont.

**Table 2:** Effect of the dietary supplementation of LEX to low-energy diets on the growth performance of broilers in each experimental group measured at different growth stages.

	Control	LEX ^1^	SEM ^2^	*p*-Value
Body weight at hatch	40.17	40.33	0.260	0.6753
0–7 days				
BW ^3^, day 7	193	195	1.883	0.3694
BWG ^3^, g	153	155	1.716	0.3567
FI ^3^, g	150	150	1.411	0.7960
FCR ^3^	0.982	0.969	0.0083	0.3159
7–14 days				
BW, day 14	542	556	4.854	0.0752
BWG, g	349	361	3.604	0.0540
FI, g	399	402	4.879	0.6921
FCR	1.143	1.114	0.0072	0.0228
14–21 days				
BW, day 21	962	1008	6.171	0.0007
BWG, g	420	452	4.901	0.0016
FI, g	602	617	3.856	0.0246
FCR	1.440	1.369	0.011	0.0019
21–28 days				
BW, day 28	1530	1602	14.798	0.0087
BWG, g	568	594	10.863	0.1338
FI, g	907	904	9.343	0.7847
FCR	1.600	1.532	0.0269	0.1117
Hatch–Catch (30 days)				
BW at catch	1581	1668	19.295	0.0133
FI, g	2250	2271	15.446	0.3725
FCR	1.464	1.399	0.0112	0.0027

^1^ Control diet supplemented with 250 g/t of a combination of lysolecithin, synthetic emulsifier, and monoglycerides; ^2^ SEM: Standard error of the mean (overall), *n* = 6 replicates per treatment (25 birds per replicate); ^3^ BW: Body weight, BWG: Body weight gain, FI: Feed intake, FCR: Feed conversion ratio.

**Table 3:** Effect of the dietary supplementation of LEX to low-energy diets on slaughter weight and carcass traits of broilers in each experimental group.

	Control	LEX ^1^	SEM ^2^	*p*-Value
Slaughter weight, g	1696	1720	17.616	0.4240
Carcass weight, g	1179	1226	11.825	0.0060
Carcass yield, %	69.54	71.48	0.672	0.0431
Wing, %	7.12	7.33	0.058	0.0123
Fillet, %	15.93	16.30	0.154	0.0971
Tender, %	3.20	3.29	0.045	0.1619
Breast meat, %	19.13	19.59	0.177	0.0724
Drum, %	10.35	10.27	0.087	0.5144
Thighs, %	17.21	17.16	0.126	0.7685
Skin, %	1.89	1.81	0.031	0.0737
Abdominal fat, %	1.30	1.14	0.046	0.0121
Gizzard, %	0.94	0.98	0.025	0.2582
Heart, %	0.54	0.56	0.015	0.3163
Liver, %	2.40	2.23	0.040	0.0040

^1^ Control diet supplemented with 250 g/t of a combination of lysolecithin, synthetic emulsifier, and monoglycerides; ^2^ SEM: Standard error of the mean (overall), *n* = 60 birds per treatment (10 birds per replicate: 5 males and 5 females).

**Table 4:** Effect of the dietary supplementation of LEX to low-energy diets on the fat and moisture content of ground deboned breast, thigh, and drumstick of broilers in each experimental group.

	Control	LEX ^1^	SEM ^2^	*p*-Value
Fat content, g/100 g	5.50	4.96	0.241	0.1390
Moisture, g/100 g	73.43	74.72	0.290	0.0078

^1^ Control diet supplemented with 250 g/t of a combination of lysolecithin, synthetic emulsifier, and monoglycerides; ^2^ SEM: Standard error of the mean (overall), *n* = 8 birds per treatment (4 males and 4 females).

**Table 5:** Effect of the dietary supplementation of LEX to low-energy diets on the jejunal morphology of broilers in each experimental group.

	Control	LEX ^1^	SEM ^2^	*p*-Value
Villus height, μm	1015.88	1248.73	40.049	0.0021
Crypt depth, μm	145.33	102.67	7.534	0.0025
Villus:crypt ratio	7.06	12.45	0.701	0.0003

^1^ Control diet supplemented with 250 g/t of a combination of lysolecithin, synthetic emulsifier, and monoglycerides; ^2^ SEM: Standard error of the mean (overall), *n* = 6 replicates per treatment (1 bird per replicate).

## Data Availability

Data are available upon request.
